# Long-Term Efficacy of Various Natural or “Green” Insecticides against Bed Bugs: A Double-Blind Study

**DOI:** 10.3390/insects5040942

**Published:** 2014-11-28

**Authors:** Jerome Goddard

**Affiliations:** Department of Biochemistry, Molecular Biology, Entomology, and Plant Pathology, Mississippi State University, Box 9775, Mississippi State, MS 39762, USA; E-Mail: Jgoddard@entomology.msstate.edu; Tel.: +1-662-325-2085

**Keywords:** bed bugs, control, insecticides, green products, natural products, IPM

## Abstract

Bed bugs are resurging throughout the world, and, thus, effective pest control strategies are constantly needed. A few studies have evaluated 25(b) and other natural, or so-called “green” products, as well as over-the-counter insecticides for bed bugs, but additional studies are needed to determine efficacy of bed bug control products. This double-blinded research project was initiated to examine long-term effectiveness of six commercially available natural or “green” insecticides against bed bugs and to compare them with three known traditional residual products. Water was used as a control. Products were evaluated against both susceptible and resistant strains of bed bugs (1200 bugs each), and two different substrates were used. Temprid^®^ (Bayer Corporation, Monheim, Germany), Transport^®^ (FMC Corp., Philadelphia, PA, USA), Invader^®^ (FMC Corporation, Philadelphia, PA USA), Cimexa^®^ (Rockwell Laboratories, Kansas City, MO, USA), and BBT-2000^®^ (Swepe-Tite LLC, Tupelo, MS, USA) were the only products which showed any substantial (>40%) bed bug control upon exposure to treated substrates after the six-month waiting period, although results with the resistant bed bug strain were much reduced. Alpine dust^®^ (BASF Corporation, Florham Park, NJ, USA) killed 27% of bed bugs or less, depending on strain and substrate. EcoRaider^®^ (North Bergen, NJ, USA) and Mother Earth D^®^ (Whitmire Microgen, Florham Park, NJ, USA) (diatomaceous earth) produced 11% control or less. Cimi-Shield Protect^®^ (Pest Barrier, Carson, CA, USA) showed no activity against bed bugs in this study. Analysis using SAS software showed a three-way interaction between treatment, substrate, and bed bug strain (Numerator DF 9; Denominator DF 80; F = 4.90; *p* < 0.0001).

## 1. Introduction

Bed bugs are blood-feeding pests of various warm-blooded animals, such as humans, bats, birds, and pets [1,2,3,4]. These parasites had nearly disappeared in developed countries until fairly recently, when a dramatic increase and spread of the insects began in the 1990s [5,6]. Since then, bed bugs have been increasingly reported inside U.S. hotel rooms, dorms, and apartments [7,8,9]. Health effects from bed bug bites include pruritic lesions and rashes, bullae, and, rarely, systemic allergic reactions [6,10,11,12,13,14,15]. There is currently little evidence supporting significant disease transmission by bed bugs [16]. Pest control of bed bugs is a multi-million dollar industry, and many products are heavily promoted as effective; however, objective data and evidence are often lacking. Many natural pesticides qualify for exemption under section 25(b) of the Federal Insecticide, Fungicide and Rodenticide Act (FIFRA) and therefore are not required to provide efficacy data for registration. A few laboratory and field studies have been undertaken to evaluate 25(b) and other natural, or so-called “green” products, as well as over-the-counter insecticides for bed bugs [17,18,19,20], but additional studies are needed. This project was initiated to examine the long-term effectiveness of several commercially available natural or “green” insecticides against bed bugs.

## 2. Experimental

### 2.1. Bed Bugs and Substrates

This work was performed in the Department of Biochemistry, Molecular Biology, Entomology, and Plant Pathology, Mississippi State University, between 26 February, 2014, and 15 August, 2014. Recently fed susceptible and resistant strains of bed bugs were used in this study (provided by Dr. Ken Haynes at the University of Kentucky, Lexington, KY, USA)—the Ft. Dix, Harold Harlan strain (susceptible), and a combination of FF1 and CIN10 field strains (resistant). Bed bugs from two resistant strains had to be combined due to the large number of bed bugs needed for this test. As for this stage, we used a combination of mixed-sex adults and late-stage nymphs. Two substrates were treated with the products being evaluated: (1) upholstery fabric (Hancock Fabrics, Tupelo, MS, USA) cut into 8 cm diameter circles as a “soft” substrate, and 4.5 cm square ceramic tiles (Lowe’s Inc., Mooresville, NC, USA) as a “hard” substrate.

### 2.2. Insecticides Tested

Six natural or “green” insecticides were chosen for this study, along with 3 traditional residual products as standards for comparison (Table 1). Note: 1.0% propoxur (Invader^®^) was included as one of the standards even though it is not appropriately labeled for bed bug control. Water was used as a control. All products were either purchased commercially or donated by manufacturers or distributors. Each product was mixed exactly per label directions, or applied “as is” if it was a ready-to-use or an aerosol product.

**Table 1:** Insecticides used in the study.

Product	% Active ingredients	How Applied
Natural, 25(b), or Green Products		
Alpine dust (BASF Corp., Florham Park, NJ, USA)	95.0% diatomaceous earth	0.32 oz per 10 ft^2^
	0.25% dinotefuran	
BBT-2000 (herbal product; primarily cedar and soybean oil) (Swepe-Tite LLC, Tupelo, MS, USA)	4.0% cedar oil	Per label, “as a fine mist”
	1.0% soybean oil	
Cimexa (Rockwell Labs, Kansas City, MO, USA)	100% amorphous silica gel	2 oz per 100 ft^2^
Cimi-Shield Protect (PestBarrier Inc., Carson, CA, USA)	2.38% soybean oil	Per label, “mix with distilled water, place in never-used spray container, shake vigorously, fan spray from 24 inches, 3 linear ft per second”
	13.18% calcium silicate	
	9.14% aluminum sodium silicate	
	7.52% iron oxide	
	7.38% sodium sulfate	
	7.32% magnesium silicate	
	4.20% potassium sulfate	
	2.38% sodium benzoate	
EcoRaider (Reneotech Inc., North Bergen, NJ, USA)	1.0% geraniol	Per label, “fan spray until wet”
	1.0% cedar oil	
	2.0% sodium lauryl sulfate	
Mother Earth D (Whitmire Micro-Gen, Florham Park, NJ, USA)	100% diatomaceous earth	0.5 oz per 10 ft^2^
Traditional Residual Products		
Invader (FMC Corp., Philadelphia, PA, USA)	1.0% propoxur	Per label, “crawling pests, 1 s spray per spot”
Temprid Readyspray (Bayer Corp., Monheim, Germany)	0.025% cyfluthrin and 0.050% imidacloprid	Per label for spot treatment, “spray 12 to 18 inches from surface, 4 s per 2 ft^2^”
Transport GHP (FMC Corp., Philadelphia, PA, USA)	22.73% acetamiprid	0.3 oz per gallon water per 1000 ft^2^
	27.27% bifenthrin	

### 2.3. Blinding of the Study

Neither the person(s) making the insecticide applications nor the person(s) evaluating mortality from the products knew which product was which. Blinding was conducted as follows. Diluted liquids were placed in clean, never-used, pump spray bottles (Consolidated Plastics Inc., Stow, OH, USA) and labeled A, B, C, *etc.* For aerosol products, clean white paper was taped over each can and also labeled with a letter. Dusts were placed in hand-held pump dusters (not electric) and also labeled with a letter. Two agricultural entomology graduate students with no experience or knowledge concerning bed bugs, but with extensive experience in insecticide application, were enlisted to apply the products to upholstery and tile substrates. For each product, label application instructions were written on blank 3 × 5 index card and handed to the graduate students along with the disguised product. The only verbal instruction given to the graduate students was, “apply this exactly as the instructions say.” Treated substrates were then placed in 100 mm × 15 mm standard plastic petri dishes (Fisher Scientific, Waltham, MA, USA), labeled A, B, C, *etc.* and their replication number, and allowed to dry. The author observed all insecticide applications to ensure they were made according to label; however, after the dishes were randomized and re-numbered (see below), even the author did not know which products corresponded to the various treatments until the very end of the study after all evaluations were complete. Three weeks after insecticide application, another entomology graduate student was enlisted to completely erase the previous labels, randomize them, re-label them with numbers 1, 2, 3, *etc.*, and hide the written list. Six months after treatment, the author exposed bed bugs to the various pre-treated substrates (see next Section) for 24 h, and after an additional 24 h, a graduate student from the School of Veterinary Medicine was enlisted to evaluate all bed bugs as to “alive” or “dead.” Results were given back to the entomology graduate student who then translated which product corresponded to which treatment/control.

### 2.4. Storage of Treated Substrates

**Figure 1:**
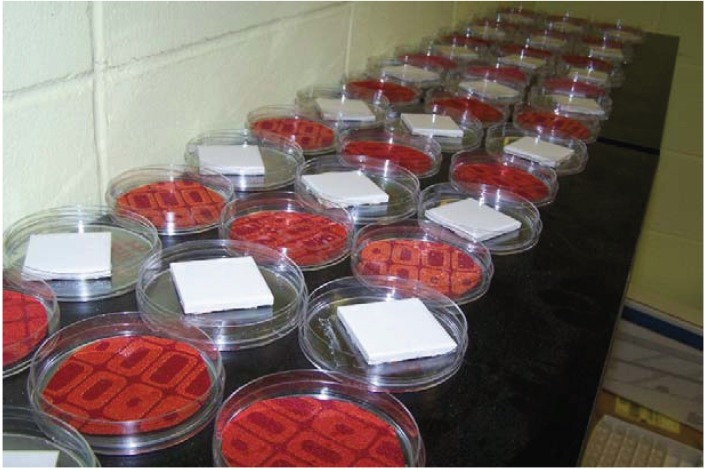
Both soft and hard substrates were treated with insecticides and stored uncovered for 3 months, then covered for 3 more months.

After tiles and upholstery pieces were treated with various products, they were placed uncovered (petri dish tops placed underneath each dish) on a shelf in the lab for 3 months to simulate “open air” field application scenarios (Figure 1), and then, petri dish tops were replaced for an additional 3 months. During the entire 6 month period, petri dishes were exposed to normal light/dark cycles of a typical laboratory 40 h work week at approximately 22 °C and 50% humidity. There were no windows in the lab, and, thus, no exposure to sunlight.

### 2.5. Exposure of Bed Bugs to Insecticides

**Figure 2:**
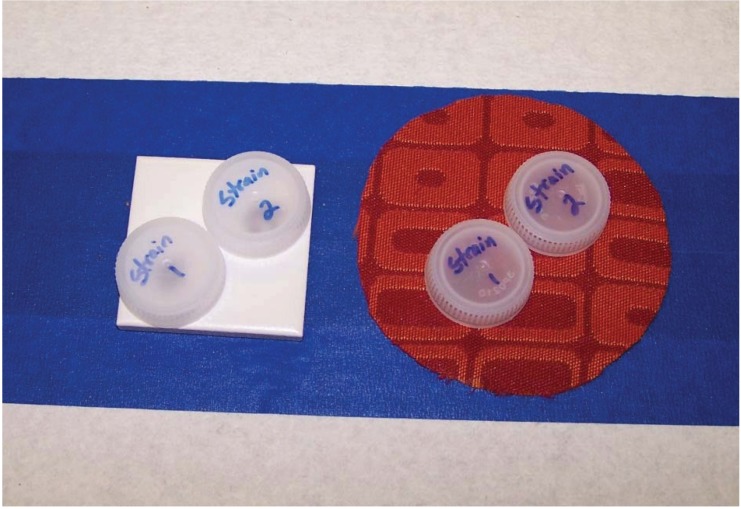
Bottle caps were used to expose bed bugs of two different strains to each treatment simultaneously.

Both the susceptible strain and resistant strain bed bugs were exposed to pre-treated substrates at the same time using bottle caps (Figure 2). Twenty bed bugs (mixed late-stage nymphs and adults) in each cap were confined on the substrates for 24 h of continuous exposure. Note: Although bottle caps contained “screw” threads which theoretically could provide harborage and discourage movement to the treated surface, after the 24 h exposure period, numerous bed bugs (regardless of treatment type) invariably were seen resting directly on the pre-treated substrates. Three replicates each of 9 treatments and 1 control × 2 strains × 2 substrates were exposed to the insecticides, yielding a total of 2400 bed bugs tested. After exposure to the various pre-treated substrates, bed bugs were placed in clean petri dishes containing filter paper for another 24 h (total of 48 h from initial exposure) and then observed for mortality. Bed bugs with no leg movement were considered dead. Results were recorded and entered into an Excel spreadsheet.

### 2.6. Statistical Analyses

Data in this study were analyzed using PROC MIXED in SAS software [21]. Data for number of bed bugs remaining alive after exposure were analyzed to determine whether they were a function of treatment, substrate, bed bug strain, or combinations of each.

## 3. Results and Discussion

**Figure 3:**
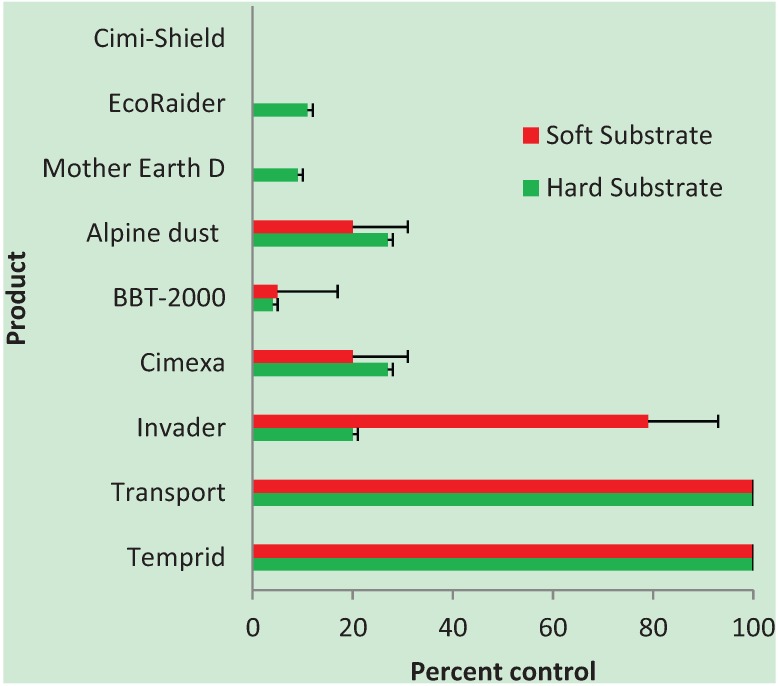
Efficacy of selected insecticides against a susceptible bed bug strain, all reps combined.

**Figure 4:**
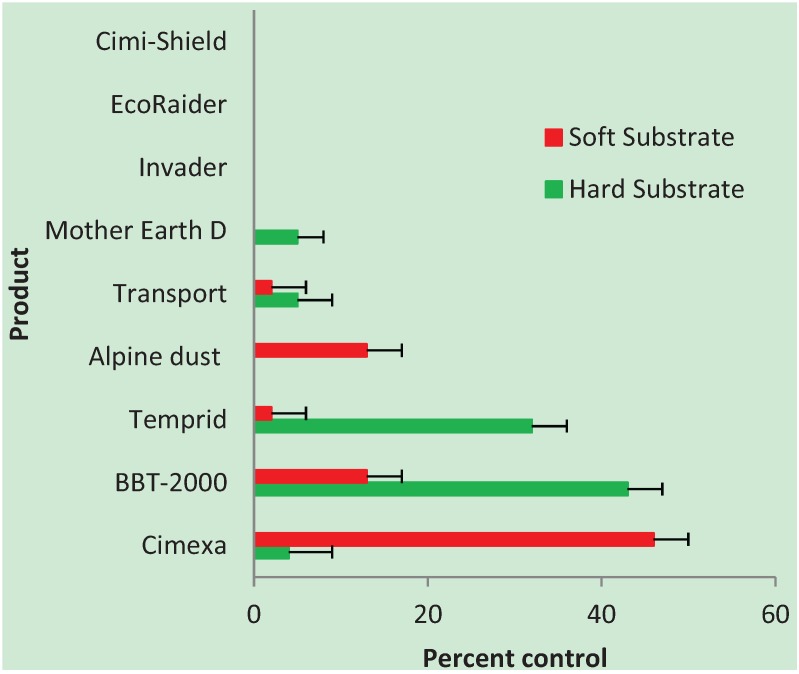
Efficacy of selected insecticides against a resistant bed bug strain, all reps combined.

Sixteen of the 240 (6.6%) control bed bugs were dead at 24 h after treatment (the time of evaluation for all bugs); therefore, for percent control calculations, our results were adjusted using the Abbott correction [22]. Temprid^®^, Transport^®^, Invader^®^, Cimexa^®^, and BBT-2000^®^ were the only products which showed any substantial (> 40%) bed bug control upon exposure to pre-treated substrates after the six-month waiting period, although results with the resistant bed bug strain were much reduced (see Section below) (Figure 3 and Figure 4). Alpine dust^®^ killed 27% of bed bugs or less, depending on strain and substrate (Figure 3 and Figure 4). EcoRaider^®^ and Mother Earth D^®^ showed only 11% control or less. Cimi-Shield Protect^®^ showed no activity whatsoever against bed bugs in this study. It should be noted that diatomaceous earth products (such as Alpine dust^®^ and Mother Earth D^®^) may take several days to produce bed bug mortality [23].

Further analysis indicated a three-way interaction between treatment, substrate, and bed bug strain (Numerator DF 9; Denominator DF 80; F = 4.90; *p* < 0.0001). In that analysis, only Transport^®^, Temprid^®^, Invader^®^, Cimexa^®^, BBT-2000^®^ and Alpine dust^®^ were significantly different from the water control (Table 2). Some possible reasons why the other three products showed no difference from water controls could be bed bug deaths in those reps due to handling (all 2400 bed bugs were individually picked up with forceps and placed in petri dishes) or possibly not enough time allowed after exposure for death to occur. However, handling deaths theoretically would be the same for insects used in controls and treatments, and there were 48 h between the time of initial exposure and evaluation.

**Table 2:** Statistical analysis of treatment × substrate × bed bug strain.

Treatment	Substrate	Strain	Mean	Standard Error	Letter Group
Transport	Hard	Susceptible	100.00	0	A *
Transport	Soft	Susceptible	100.00	0	A
Temprid	Hard	Susceptible	100.00	0	A
Temprid	Soft	Susceptible	100.00	0	A
Invader	Soft	Susceptible	80.00	17.56	B
Cimexa	Soft	Resistant	50.00	2.89	C
BBT-2000	Hard	Resistant	46.67	17.64	CD
Temprid	Hard	Resistant	36.67	10.93	CDE
Cimexa	Hard	Susceptible	31.67	19.65	DEF
Alpine dust	Hard	Susceptible	31.67	7.27	DEF

* Different letters indicate significant differences.

### 3.1. Susceptible Strain

Both Temprid^®^ and Transport^®^ killed 100% of susceptible bed bugs when placed on pre-treated hard and soft substrates after the six-month time period (Figure 3). Invader^®^ killed 79% of susceptible bugs on the upholstery fabric (soft), but only 20% on tiles (hard). Both Cimexa^®^ and Alpine dust^®^ produced 27% and 20% control of susceptible bed bugs on hard and soft substrates, respectively. The other products showed little activity.

### 3.2. Resistant Strain

None of the products tested demonstrated >50% efficacy against the resistant strain of bed bugs. This is not surprising in light of recent studies demonstrating that insecticide resistance is increasing in field populations of bed bugs, even to the successful dual action products, such as Temprid^®^ and Transport^®^ [24,25,26]. However, Cimexa^®^ killed approximately half of resistant bugs on the upholstery fabric, while Temprid^®^ killed 32% on ceramic tiles (Figure 4). As for why Cimexa^®^ (100% amorphous silica gel) seemed ineffective on the tiles, perhaps the powder did not stick to the slick tile surface. Silica gel particles are so lightweight they often suspend in the air following application [26]. Interestingly, the natural product, BBT-2000^®^, composed primarily of cedar and soybean oil, killed 43% of resistant bed bugs on pre-treated tiles, a better result than either Temprid^®^ or Transport^®^. In a previous study, this same product, formerly labeled as Swepe Tite Bed Bug Treatment^®^, produced 70% control of bed bugs when exposed to ceramic tiles which had been treated 24 h earlier [27], thus, there is some evidence that these natural oily products can have a residual effect.

In their defense, some natural or green products do not claim to kill bed bugs months after treatment. In fact, many of them simply claim to “kill bed bugs on contact.” In this study, we did not test at shorter time intervals to determine the residual life of these products. However, some products do indeed claim a residual effect. Cimexa^®^ claims that the product lasts up to 10 years if undisturbed, which implies that it would still have activity against insect pests for that time period, and recent field tests with the product have shown great promise [26]. EcoRaider^®^ claims to provide “long-lasting” protection but does not define how long that is. The BBT-2000^®^ label says, “repeat every 1–2 weeks for prevention,” which implies a residual effect for that time period. Mother Earth D^®^ (diatomaceous earth) says on its labeling, “provides long-lasting control of insect pests.” On a website, Cimi-Shield Protect^®^ specifically claims to “prevent bed bug infestations for a full year.” This particular claim seems in stark contrast to the data presented here.

There are places and times where natural or green insecticides are warranted for bed bug control, such as in sensitive accounts like classrooms, day care centers, nursing homes, certain areas of hospitals, and the like. However, pest management professionals and homeowners alike should realize that no one product is a magic bullet and an integrated approach to bed bug management is always best.

## 4. Conclusions

Although many natural or “green” insecticides are heavily promoted on the Internet as effective bed bug control products, careful and objective evaluation of these products is often lacking. In this study, the only natural or “green” products producing any substantial bed bug control upon exposure to pre-treated substrates after a six-month waiting period were BBT-2000^®^, Cimexa^®^, and to a lesser extent, Alpine dust^®^.
